# Survival of patients with alcoholic and cryptogenic cirrhosis without liver transplantation: a single center retrospective study

**DOI:** 10.1186/1756-0500-5-663

**Published:** 2012-12-02

**Authors:** Sudul Mananjala Senanayake, Madunil Anuk Niriella, Sanjaya Kumara Weerasinghe, Anuradhani Kasturiratne, Jerome Praneeth de Alwis, Arjuna Priyadarsin de Silva, Anuradha Supun Dassanayake, Hithanadura Janaka de Silva

**Affiliations:** 1 University Medical Unit, Colombo North Teaching Hospital, Ragama, Sri Lanka; 2 Departments of Medicine, Ragama, Sri Lanka; 3 Departments of Public Health, Ragama, Sri Lanka; 4 Departments of Pharmacology, Faculty of Medicine, University of Kalaniya, Ragama, Sri Lanka

**Keywords:** Alcoholic cirrhosis, Cryptogenic cirrhosis, Transplant-free survival

## Abstract

**Background:**

There is no recent data addressing the long term survival of cirrhosis patients without transplantation, but with the availability of optimal pharmacological and endoscopic therapies. We compared the long term transplant free survival of alcoholic (AC) and cryptogenic (CC) cirrhosis patients in a setting where liver transplantation was, until very recently, not available. AC and CC patient details were extracted from our database, maintained since 1995. For those who had not attended clinics within the past 4 weeks, the patient or families were contacted to obtain survival status. If deceased, cause of death was ascertained from death certificates and patient records. Survival was compared using Kaplan-Meier curves.

**Results:**

Complete details were available in 549/651 (84.3%) patients (AC 306, CC 243). Mean follow up duration (SD) (months) was 29.9 (32.6). 82/96 deaths (85.4%) among AC and 80/94 deaths (85.1%) among CC were liver related. Multivariate analysis showed age at diagnosis and Child’s class predicted overall survival among all groups. The median survival in Child’s class B and C were 53.5 and 25.3 months respectively. Survival was similar among AC and CC. Among AC survival was improved by abstinence [HR = 0.63 (95% CI: 0.40-1.00)] and was worse with diabetes [HR=1.59 (95% CI: 1.02- 2.48)] irrespective of alcohol status.

**Conclusions:**

The overall survival of AC was similar to CC. Death in both groups were predominantly liver related, and was predicated by age at diagnosis and Child class. Among AC, presence of diabetes and non-abstinence from alcohol were independent predictors for poor survival.

## Background

The long term survival of cirrhotic patients is an important issue in management, from the initial counseling of the patients at diagnosis to finally planning liver transplantation [1]. This becomes a central issue in settings where liver transplantation is not readily available. Decompensated cirrhosis has a dismal prognosis without transplantation. Although older studies in the pre-transplantation era have used measures such as the Child-Pugh score to predict survival [2,3], new pharmacological and endoscopic therapies have had a significant impact on improving patient survival [4,5]. As liver transplantation has becomes a viable option, even in many developing countries, it has become increasingly difficult to study the natural history of the disease. Although there have been published studies of survival in cirrhosis, these have been on selected populations suitable for transplant or with single aeitology or with relatively short follow up periods [6-8]. Although prospective studies are more valid to assess long term mortality, they would be impossible to perform in the current context as liver transplant becomes increasingly available. Therefore even retrospective data from centers where patients have until very recently not had the option of liver transplantation, may provide valuable information on the natural history of cirrhosis with the currently available pharmacological and endoscopic therapies.

Liver transplantation is in its infancy in Sri Lanka. Until recently only very few patients with the means of getting the procedure performed abroad had a liver transplant. It is still not widely available for most cirrhotic patients attending state sector hospitals. This has provided an opportunity to study the natural history of the disease in a setting where patients have the benefit of most modern therapies, with the exception of transplantation. The main causes of cirrhosis in Sri Lanka are alcohol induced and cryptogenic. The long term comparative survival of these two causes of cirrhosis has been poorly studied especially during the last twenty years [9-12]. We report our findings where we studied and compared the long term survival of alcoholic (AC) and cryptogenic (CC) cirrhosis patients who did not have the option of liver transplantation.

## Methods

The study was conducted in the Gastroenterology Clinic of the University Medical Unit, Colombo North Teaching Hospital, Ragama, Sri Lanka, which serves as a major referral center for cirrhosis. Details of all cirrhotic patients in our clinic have been maintained since 1995.

### Study population

All patients diagnosed with AC and CC registered in our clinic were eligible for inclusion in the study. Cirrhosis was diagnosed on clinical, biochemical, abdominal imaging, and endoscopic criteria and, when possible or required, confirmed by liver biopsy. Patients who were found to have hepatocellular carcinoma at diagnosis were excluded from the study. AC had a history of consuming alcohol above the accepted safe limits (Asian standards: <14 units alcohol per week in men and <7 units per week alcohol in women) prior to the diagnosis of cirrhosis. CC were patients who did not drink alcohol above the safe limit, had no history of contributory drug or herbal product use and in whom Hepatitis B and C, autoimmune disease, haemachromatosis, Wilson’s disease, and alpha-1 antitrypsin deficiency were excluded. Patients in whom cirrhosis was diagnosed prior to availability of Hepatitis C testing were subjected to the test once it became available. Therefore, all patients attending the clinic had their Hepatitis B and C status assessed at some time during follow-up.

### Study design

This was a retrospective cohort study. Patients diagnosed with AC and CC registered in our clinic since January 1995 were included. This study was conducted over a period of 4 weeks in June 2010. Patients who had not attended the clinic within 4 weeks of the study were identified, and either the patients or their families were contacted by telephone or post to obtain survival status, and if deceased, details of death. The cause of death was confirmed from death certificates and patient records. Clinical details of AC and CC were obtained, and included the date of diagnosis of cirrhosis, liver biochemistry with INR and Child Pugh grade at diagnosis.

Patients with AC and their families were interviewed for details of alcohol consumption following the diagnosis of cirrhosis. ‘Alcohol abstinence’ was defined as patents who claimed that they never consumed alcohol, following the diagnosis of cirrhosis. Details of abstinence were confirmed from family members after consent from the patient. If the individual patient or the family claimed that alcohol (of any quantity) was consumed on one or more occasions following diagnosis of cirrhosis, the patient was defined as ‘non – abstinent’.

In those whom this information was available, abstinence or non-abstinence for alcohol was recorded. The presence of diabetes anytime during the follow up period was recorded in all patients.

### Ethics approval

Prior ethics approval for this study was obtained from the Ethics Review Committee of the Faculty of medicine, University of Kelaniya. Informed consent was taken from all participating patients and their families regarding obtaining information as well as follow up data.

### Statistical analysis

Unadjusted and adjusted hazard ratios and 95% confidence intervals were estimated using Cox’s proportional hazards modeling for the association between mortality and associated factors. Potential confounding variables considered in the model were age, sex, BMI, diabetes mellitus, Child-Pugh Grade at diagnosis and the aetiology of cirrhosis (AC or CC). All potential confounding variables were included in the multivariate model.

Survival was compared using Kaplan-Meier curves using SPSS (version 16.0) and predictors of survival in each group were sought. The mean and median survival of cirrhotics was calculated according to Child’s Pugh class.

## Results and discussion

Of the 696 registered cirrhotic patients, AC and CC accounted for 651 (93.53%) (Table 1). The numbers of patients having cirrhosis due to other aetiologies were considered too small to study their natural history. Furthermore, the possibility of at least some patients defined as CC being surreptitious drinkers, and therefore in reality being AC should be considered when interpreting these data.

**Table 1:** Aetiology of cirrhosis in database

**Aetiology**	**Number**	**Percentage**
**Alcohol (AC)**	**381**	**54.74%**
**Cryptogenic (CC)**	**270**	**38.79%**
Hepatitis B	13	1.87%
Wilson’s disease	13	1.87%
Autoimmune	7	1.01%
Drug induced	4	0.57%
Hepatitis C	3	0.43%
Haemochromatosis	3	0.43%
PBC	1	0.14%
Traditional and herbal medicines	1	0.14%
**Total**	**696**	**100.00%**

Of the 651 patients with AC or CC, complete details were available for analysis in 549 (84.3%) (AC 306, CC 243). Demographic and clinical characteristics in these patients are given in Table 2. Type 2 diabetes or obesity was present in 68.7% of patients with CC. Details of AC and CC with and without follow up data are given in Table 3.

**Table 2:** Selected demographic, clinical data and deaths among study population

	**Alcoholic Cirrhosis (n=306)**	**Cryptogenic Cirrhosis (n=243)**	**Total (n=549)**
Mean age (SD) at diagnosis (years)	52.5 (9.7)	58.4 (10.6)	55.1 (10.5)
Sex	Male :299	Male :135	Male :434
	Female :7	Female :108	Female :115
Child class at diagnosis	A :74 (24.18%)	A :87 (35.80%)	A :161 (29.33%)
	B :159 (51.96%)	B :125 (51.44%)	B :284 (51.73%)
	C :73 (23.86%)	C :31 (12.76%)	C :104 (18.94%)
Serum Bilirubin (SD)	3.3 (3.6)	2.3 (1.9)	2.9 (3.0)
Serum Albumin (SD)	29.8 (7.3)	32.7 (9.0)	31.1 (8.2)
Mean follow up duration (SD) (months)	29.25 (32.52)	30.76 (32.83)	29.92 (32.64)
No. with diabetes(Dm)	125(40.85%)	128(52.67%)	253(46.08%)
No. with obesity(BMI>=25)	68(22.22%)	71(29.22%)	139(25.32%)
Diabetes(Dm) and Obesity(BMI>=25)	Dm only:101(33.01%)	Dm only:96(39.51%)	Dm only:197(35.88%)
	Obesity only:44(14.38%)	Obesity only:39(16.05%)	Obesity only:83(15.12%)
	Both:24(7.84%)	Both:32(13.17%)	Both:56(10.20%)
	None:137(44.77%)	None:76(31.28%)	None:213(38.80%)
**Survival status**			
Alive	210(68.63%)	149(53.07%)	359(61.75%)
**Died**	**96(31.37%)**	**94(38.68%)**	**190(34.61%)**
Total	306(100%)	243(100%)	549(100%)
**Cause of death**			
**Liver related**	**82(85.42%)**	**80(85.11%)**	**162(85.26%)**
Non-liver related	14(14.58%)	14(14.89%)	28(14.74%)
Total deaths	96(100%)	94(100%)	190(100%)

**Table 3:** Details of demographic and clinical characteristics among AC and CC with and without follow up data

	**Follow up data available (n=549)**	**Follow up data not available (n=102)**
Mean age (SD) at diagnosis (years)	55.1 (10.5)	52.2 (14.6)
Sex	Male :434	Male : 92
	Female :115	Female :10
AC	306	64
CC	243	38
Child class at diagnosis	A :161 (29.33%)	A :36 (35.29%)
	B :284 (51.73%)	B : 40 (39.22%)
	C :104 (18.94%)	C : 26 (25.49%)
No. with diabetes (Dm)	253(46.08%)	35 (34.31%)
No. with obesity (BMI>=25)	139(25.32%)	28 (24.51%)

Deaths among both AC and CC were predominantly liver related (85.3%). There were 96 deaths [82 (85.4%) liver related] in the AC group and 94 deaths [80 (85.1%) liver related] in the CC group (Table 2). Among the patients with liver related deaths, causes of death were hepatic encephalopathy (37.2%), upper GI bleeding (32.6%), hepato-renal syndrome (18.6%), development of hepatocellular carcinoma in (9.3%) and spontaneous bacterial peritonitis in (2.3%).

**Figure 1:**
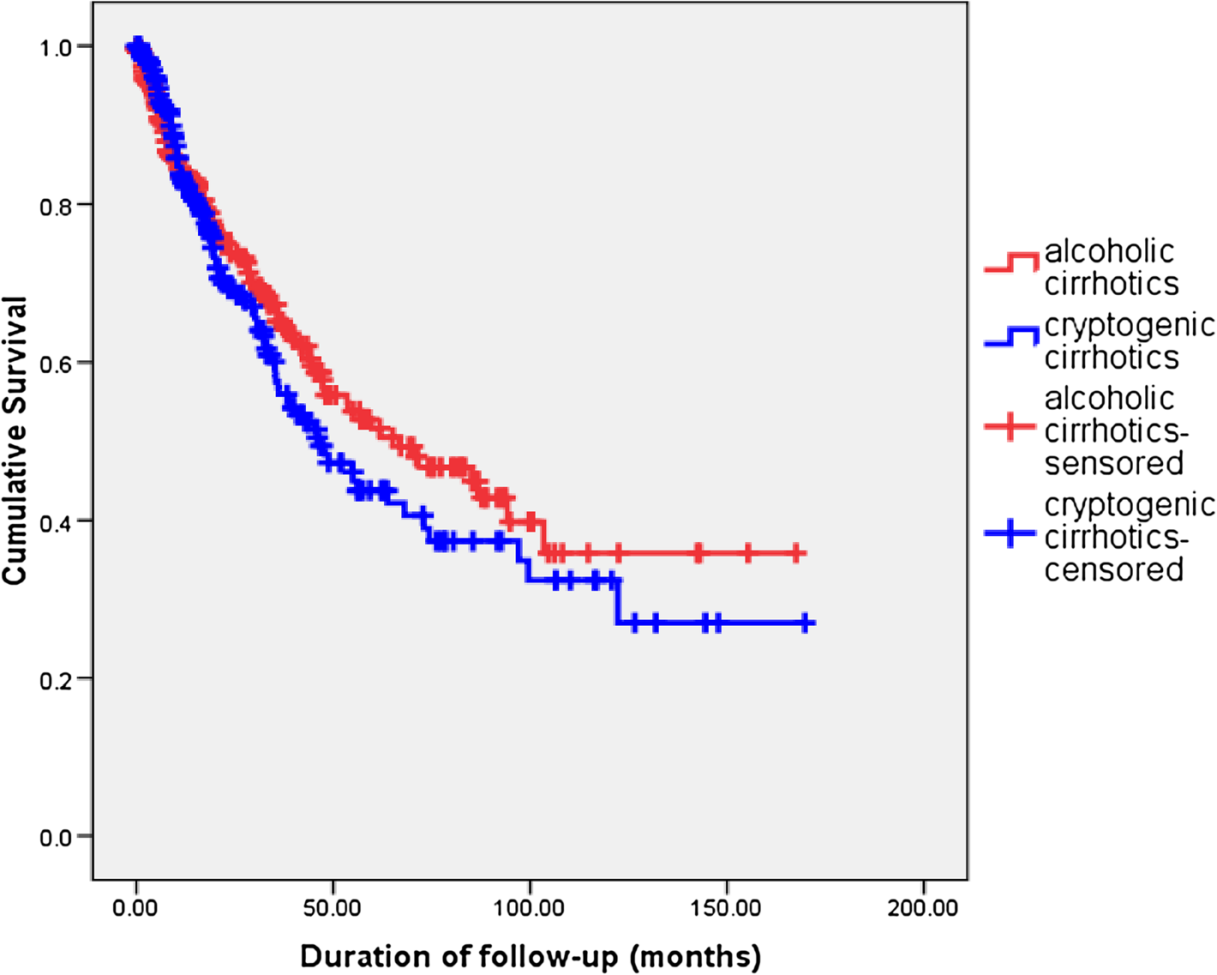
Comparison of long term survival - Alcoholic cirrhosis (AC) vs Cryptogenic cirrhosis(CC).

The overall median survival of patients in Child’s class B and C were 53.5 and 25.3 months respectively (Table 4). The duration of follow up of patients with Child’s class A cirrhosis was inadequate to assess the median survival in this group. The median survival of AC and CC were 66.9 and 46.2 months respectively. However, Kaplan-Meier survival curves showed no significant difference in survival between AC and CC (log rank test statistic 0.297) (Figure 1).

**Table 4:** Median survival of patients by Child’s grade

**Child class**	**No. of patients**	**No. of deaths**	**Median survival (months)**	**95% CI**
**A**	161(29.33%)	26(13.68%)	**-**	-
**B**	284(51.73%)	107(56.32%)	**53.5**	31.4– 75.7
**C**	104(18.94%)	57(30%)	**25.3**	15.6 – 35.0
**Overall**	549(100%)	190(100%)	**55.1**	41.0 - 69.1

(log rank test statistic <0.001).

**Figure 2:**
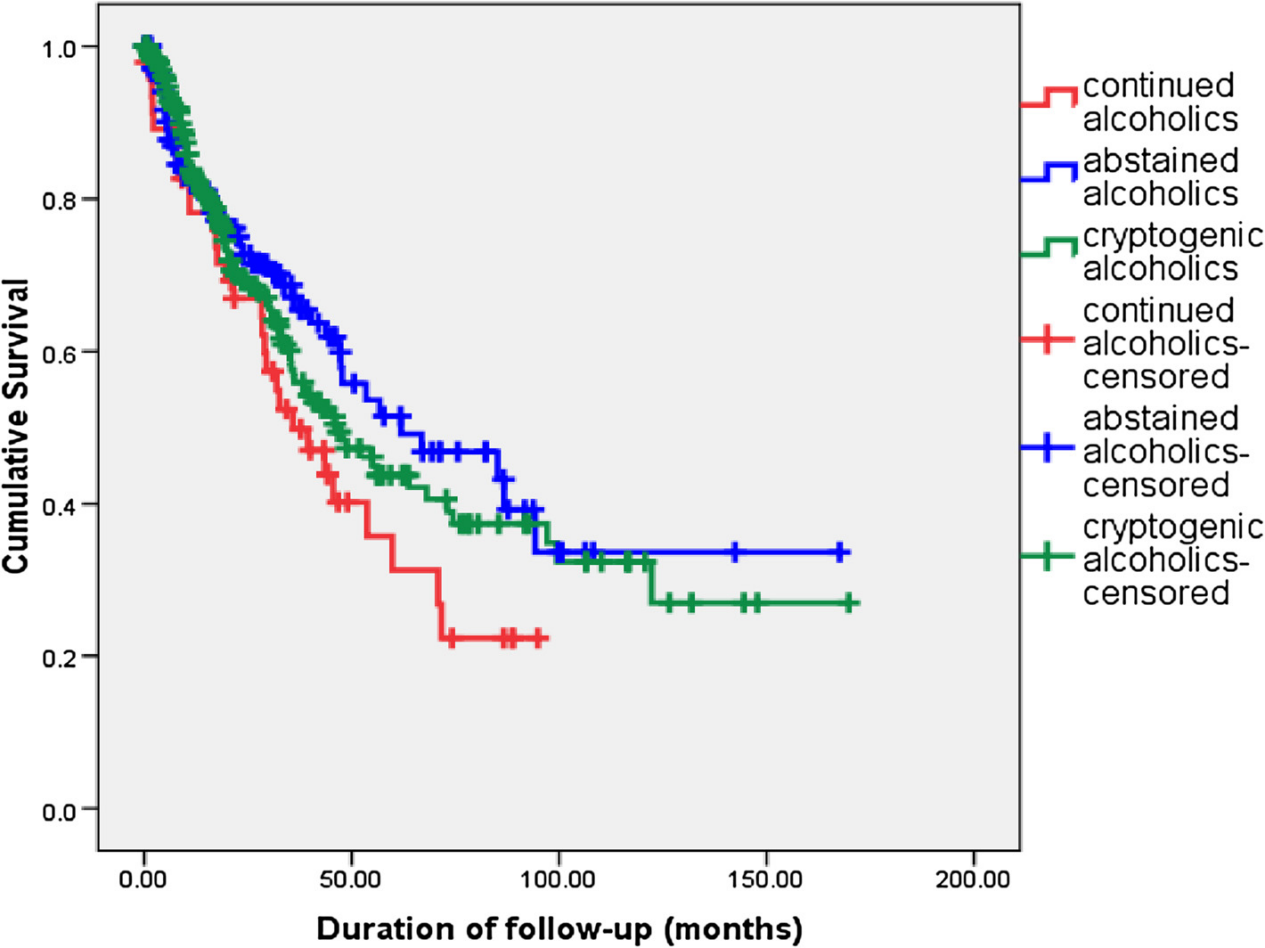
Comparison of long term survival – AC continuing alcohol vs AC claiming abstinence vs CC.

**Figure 3:**
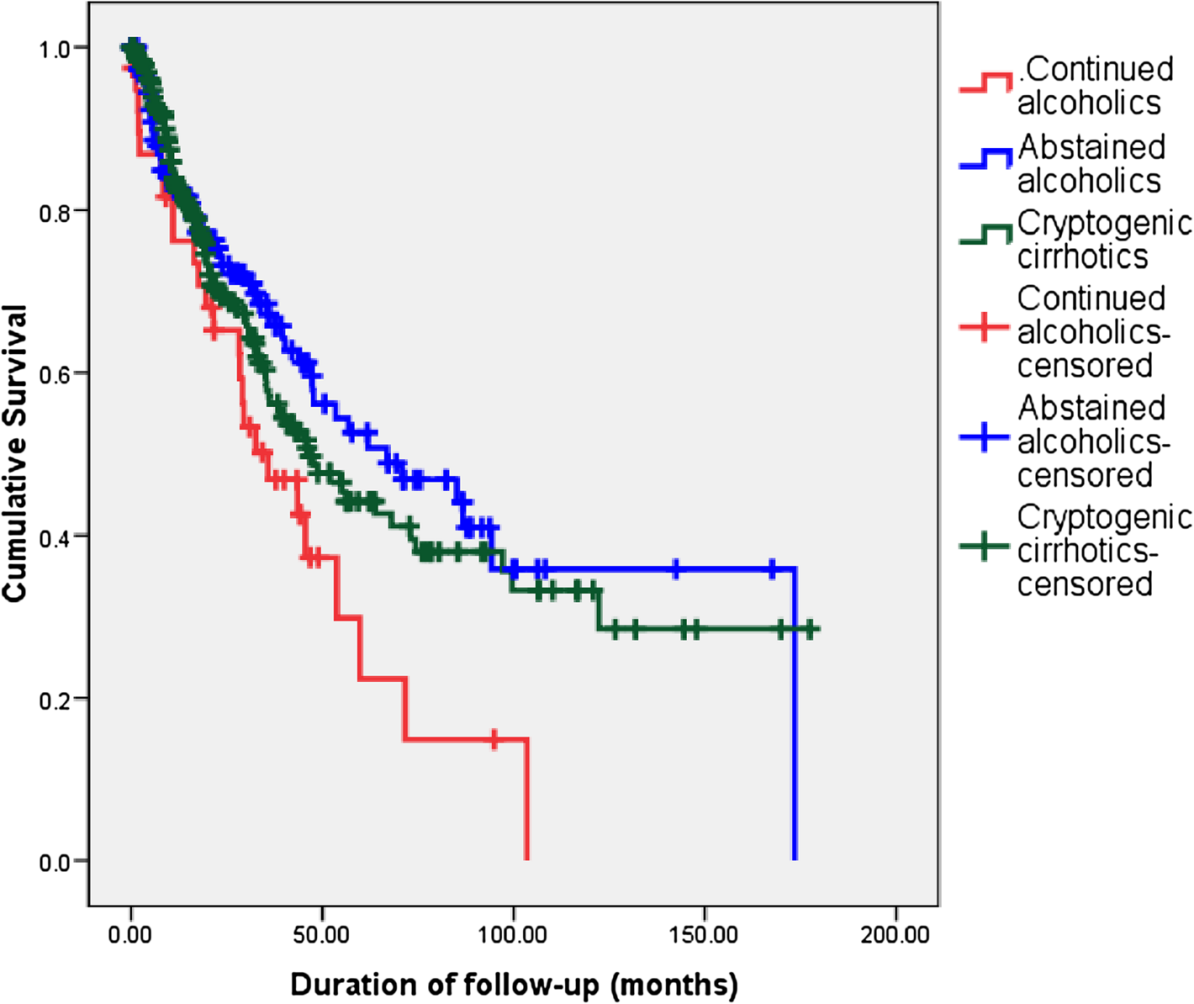
Comparison of long term survival – AC continuing alcohol at the time of diagnosis vs AC claiming abstinence at the time of diagnosis vs CC.

Among the 306 patients with AC 198 (64.7%) volunteerd information regarding alcohol consumption after diagnosis. For deceased patients this information was obtained from the family members. The percentage of AC claming to be abstinent was 75.8%. Among AC claiming abstinence (n=150), 50 patients (33.3%) had died during follow up. Among AC who were not abstaining (n=48), 30 (62.5%) had died. The survival of AC who continued to drink, AC who claimed abstinence and CC was compared (Figure 2). On comparison of Kaplan Meier survival curves using the log rank test, among AC, those who were not abstaining had a worse survival compared to AC claiming abstinence (p=0.048). Compared to CC (median survival 46.2 months), there was a trend for better survival among AC who claimed abstinence (median survival 61.9 months), however this difference was not statistically significant (p=0.476) (Figure 2). However, this data may be confounded by immortal time bias. In an attempt to overcome this we re-analysed the effects of abstinence by categorizing AC patients only according to whether they were consuming alcohol at the time of diagnosis of cirrhosis. The survival patterns of a sub-sample of AC who were continuing to drink at the time of diagnosis (n=39), AC who claimed abstinence at the time of diagnosis and CC did not change significantly from our initial analysis (Figure 3). On comparison of Kaplan Meier survival curves using the log rank test, among AC, those who were not abstaining had a worse survival compared to AC claiming abstinence. Compared to CC (median survival 46.2 months), there was a trend for better survival among AC who claimed abstinence (median survival 66.9 months), although this difference was not statistically significant.

**Figure 4:**
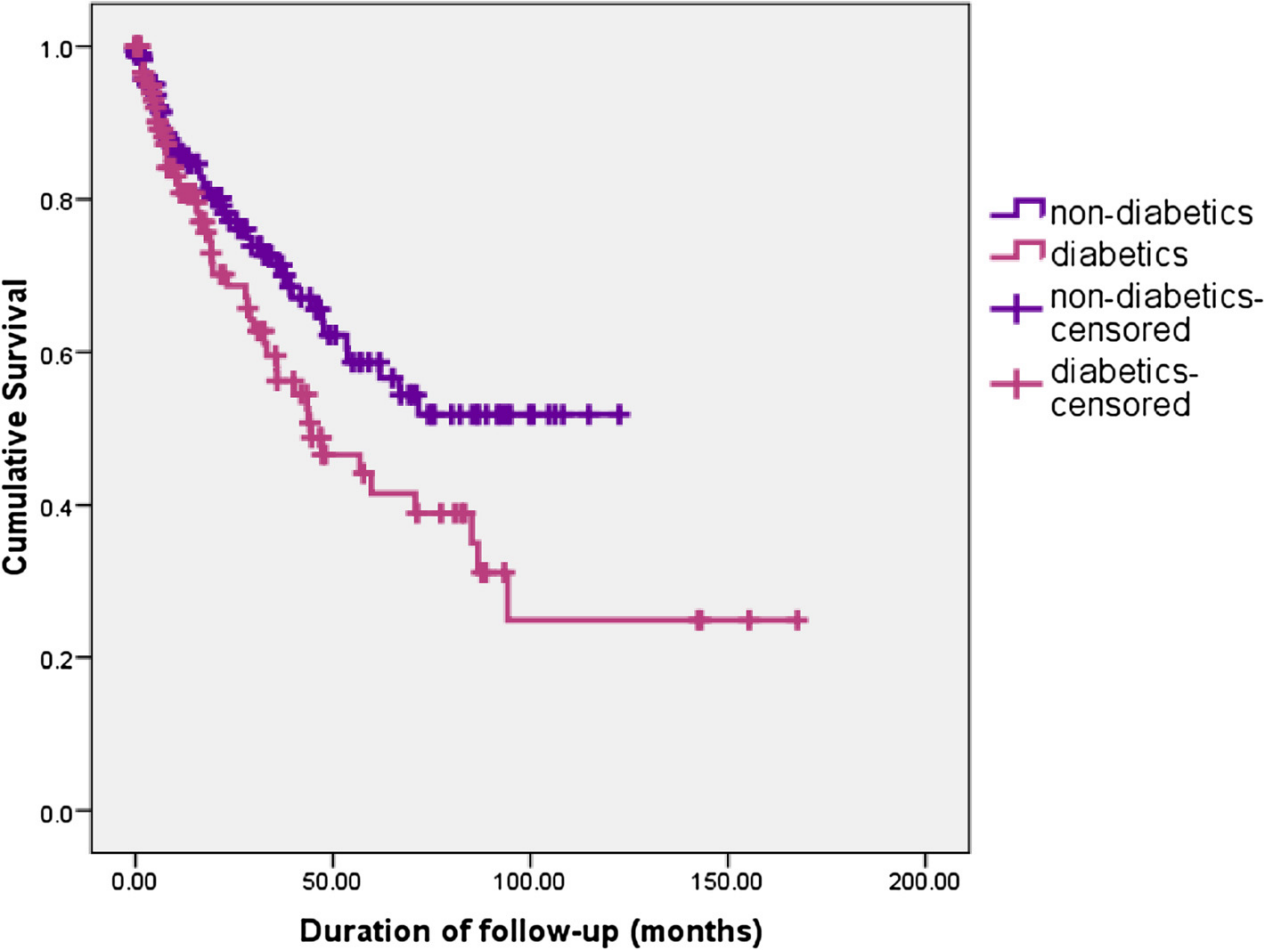
Comparison of long term survival – AC with diabetes vs AC without diabetes.

Kaplan-Meier survival curves showed a significant difference in survival between AC who did not have diabetes and those who had diabetes, with the latter having a worse prognosis (Log rank test statistic 0.024) (Figure 4). This difference persisted among AC claiming abstinance, with and without diabetes (Log rank test statistic 0.039). This adverse effect of diabetes on survival was not seen among CC (p= 0.21).

**Figure 5:**
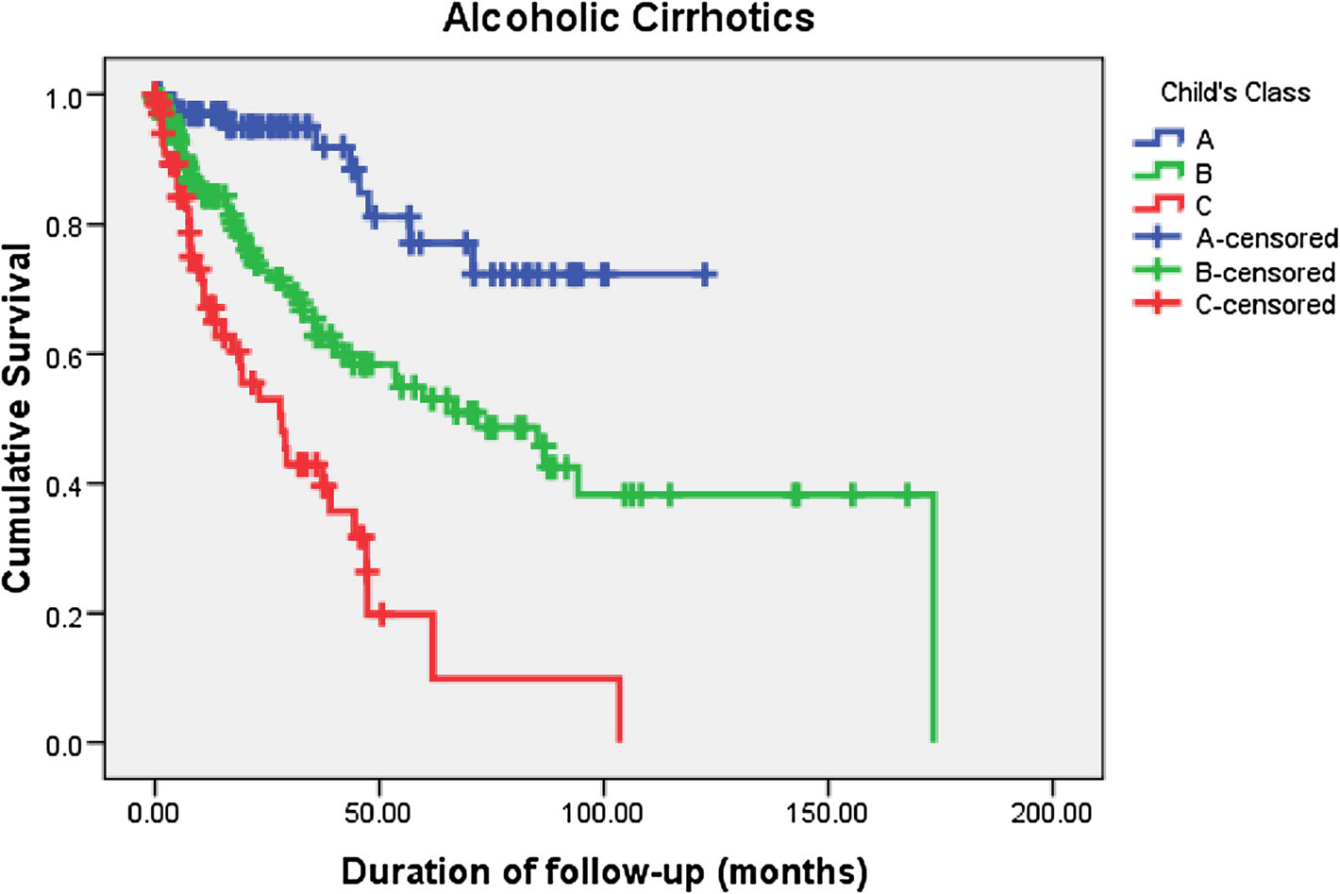
Comparison of long term survival among alcoholic cirrhotics (AC) according to Child class at diagnosis.

**Figure 6:**
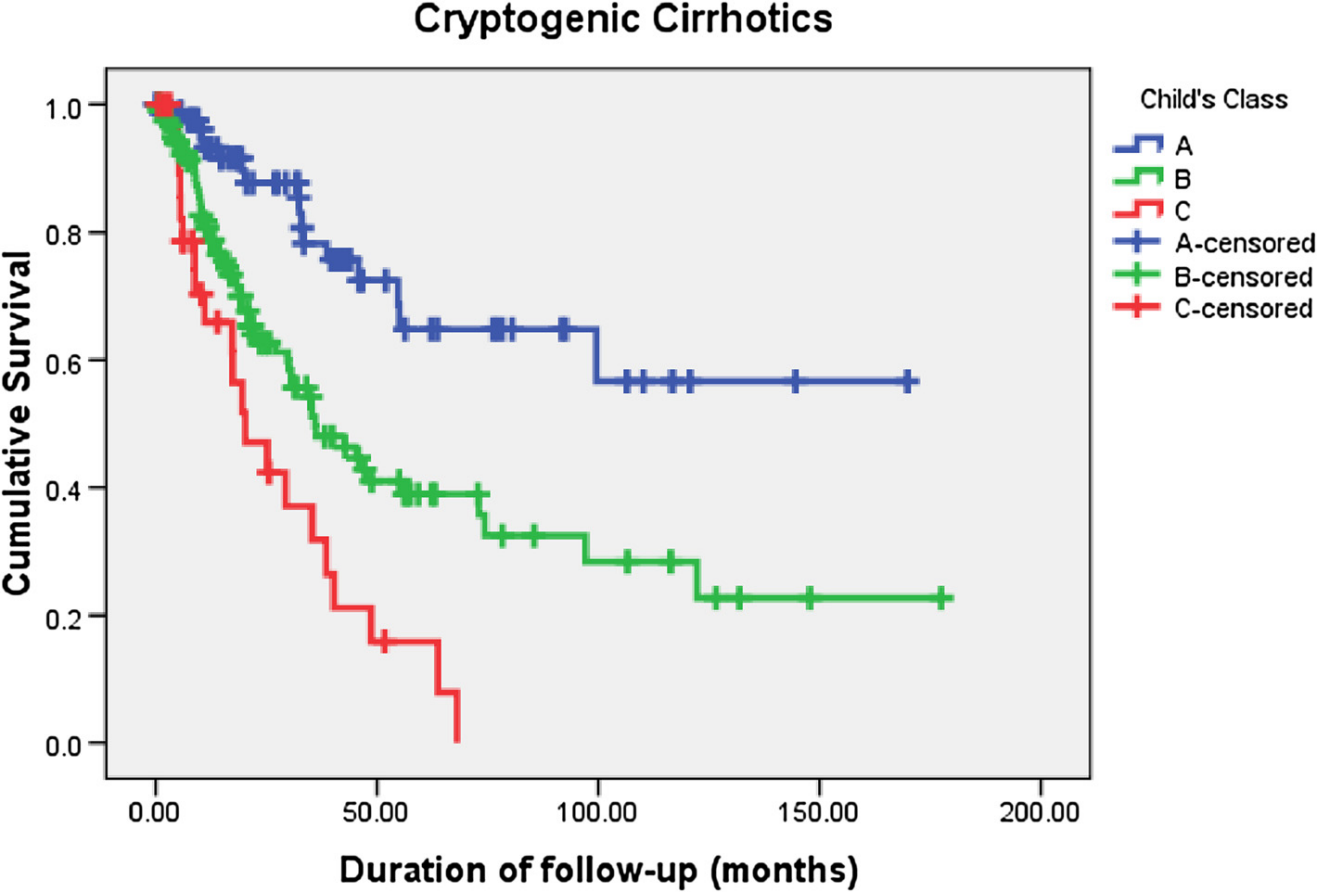
Comparison of long term survival among cryptogenic cirrhotics (CC) according to Child class at diagnosis.

On bivariate analysis, factors associated with mortality were, age at diagnosis and Child’s class (Table 5). On multivariate analysis independent predictors of mortality were: age at diagnosis and Child’s class after adjusting for sex, BMI, diabetes mellitus and aetiology of cirrhosis (AC or CC). Each year of age increased the risk of mortality by 1.03 times. A patient in Child’s class B had a 3 times higher risk of mortality as compared to a patient in Child’s class A. A patient in Child’s class C had a 7 times higher risk of mortality as compared to a patient in Child’s class A (Table 4). Kaplan-Meier survival curves for different Child’s classes among AC and CC are shown in Figure 5 and Figure 6 respectively.

**Table 5:** Factors associated with death in patients with cirrhosis

**Variable**	**Dead (n=190)**	**Alive (n=359)**	**HR (95% CI) (bivariate analysis without adjusting for any variable)**	**HR (95% CI) (multivariate analysis adjusting for all variables)**
Age (considered as a continuous variable)			1.02 (1.01-1.04)^*^	1.03 (1.01-1.04)*
BMI (considered as a continuous variable)			0.98 (0.94-1.03)	0.99 (0.96-1.04)
Male sex (%)	145 (76.3)	289 (80.5)	0.81 (0.58-1.13)	0.77 (0.48-1.25)
Child class A**	26 (13.7)	135 (37.6)	-	-
Child class B	107 (56.3)	177 (49.3)	2.83 (1.84-4.35)^*^	3.17 (1.85-5.46)^*^
Child class 3	57 (30.0)	47 (13.1)	6.21 (3.89-9.83)^*^	7.07 (3.94-12.68)*
Alcoholic cirrhosis (%)	96 (50.5)	210 (58.5)	0.86 (0.65-1.14)	0.79 (0.52-1.20)
Diabetes mellitus (%)	94 (49.5)	159 (44.3)	1.20 (0.90-1.60)	1.11 (0.78-1.56)

^*^ Significant.

** Reference category.

HR-Hazards ratio.

95% CI-95% confidence interval.

Among AC survival was improved by abstinence [HR = 0.63 (95% CI: 0.40-1.00)] and was worse in the presence of diabetes [HR=1.59 (95% CI: 1.02- 2.48)].

In our sample, none of the patients had the option of being listed for liver transplantation as this was not available in Sri Lanka at the time of the study, and none of the study population could afford transplantation abroad. Despite this, they were all provided with optimal pharmacological and endoscopic therapy. This provided an unique opportunity for us to study true transplant free survival in the current context.

Previous studies on the natural history of patients in settings where transplantation is available are fundamentally different from our study for several reasons. Firstly, patient populations of those studies comprised only of patients who qualified for a liver transplant [6,7]. Secondly, they were followed up for relatively shorter time periods [7]. Finally, the use of Kaplan –Meier survival curves is suboptimal in that situation as multiple outcomes which affect one another, namely, transplantation and death, have to be considered [6]. Kim *et al.* uses “competing risk analyses” as an alternative method of analysis, but admits that “Kaplan-Meier estimates may be the most appropriate to answer certain ‘what if’ questions, for example, when counseling a patient about their future course without transplantation” [6]. This is what our study hoped to address.

Our results indicate that the median overall survival of Child’s B and C cirrhotics was 53.5 months and 25.3 months respectively. This is in contrast to older estimates [3] which suggested a much shorter survival without liver transplantation. This may reflect better pharmacotherapy and endotherapy which is currently available altering the natural history of the disease [4,5]. This hypothesis needs further investigation.

There was a relatively short mean follow period up of 29.9 months for this study. The reason for this is that as our centre became established as a national referral centre for liver diseases the number of patients referred to it during recent times has been far greater than in the initial stages.

We found that the survival of patients with AC and CC was similar. The survival of these two groups has been poorly compared during the last twenty years. Stone *et al.* in 1968 reported that AC appeared to have a better outlook than CC [10]. Later, in 1987, Tanaka *et al.* reported that survival of AC was poorer than CC [12]. The investigators also reported that survival of both types of cirrhosis improved over the three decades of study (1958–1984) [12]. Our finding of a similar survival for both AC and CC possibly reflects overall improvement in the care of severely ill cirrhotic patients. In addition, the relatively high percentage of AC abstaining from alcohol (75.8%) in our sample, if reliable, may also have contributed to a better prognosis. The majority of deaths among AC and CC were liver related (85.3%). This is similar to earlier reports where Tanaka *et al.* reported non-liver related causes of death to be 8% [12].

We found, predictably, that abstinence from alcohol significantly improves long term survival among AC. This has been established in previous studies [11-15]. This emphasizes the importance of reinforcing alcohol abstinence in the management of these patients.

Numerous studies have reported a high prevalence of obesity and diabetes among patient with CC and suggested that most of these patients represent non alcoholic steatohepatitis (NASH) related cirrhosis [16-18]. It has been suggested that in Asia many cases of CC may be related to non alcoholic fatty liver disease [19]. Over two thirds of our patients with CC (68.7%) had diabetes or obesity. Such a high prevalence of metabolic risk factors may not be surprising among South Asian populations [20,21]. However this high percentage of patients with metabolic risk factors (diabetes or obesity) among our patients diagnosed with CC suggests that the likely aetiology of their ‘cryptogenic’ cirrhosis is non- alcoholic steatohepatitis (NASH).

In our study, diabetes was found to be an independent predictor of mortality among AC, but not among CC. Diabetes may be an aggravating factor in the evolution of cirrhosis or may occur as a consequence of cirrhosis - termed “hepatogenous diabetes” [22,23]. Although the available data is limited, it had been suggested that diabetes is associated with a worse prognosis in cirrhosis [24-26] and that impaired oral glucose tolerance may predict long term [27] as well as the short term mortality [28] among cirrhotics. However, Chen *et al.* recently reported no association between diabetes and the in–hospital mortality of cirrhosis patients, although they too found that a significant proportion of cirrhotic patients had diabetes [29]. The association between cirrhosis and diabetes, especially in relation to different aetiologies of cirrhosis, warrants further investigation. We found that diabetes continues to be a predictor of mortality even among AC who abstain from alcohol. Moreau *et al.* also reported a similar association between diabetes, abstinence and mortality among cirrhotics who had refractory ascites [25].

Demographically, the two groups of patients in our study were similar except that there were far fewer females with AC than CC. This is representative of the drinking habits in an average Sri Lankan population where, for possible cultural reasons, heavy alcohol consumption among females is very uncommon. However sex was not a significant predictor of survival when other factors (Child’s class, alcohol abstinence, presence of diabetes, obesity and the etiology of cirrhosis etc.) were adjusted for, and hence did not seem to affect survival in these two groups.

Among co-morbidities only diabetes and obesity were assessed as predictors of survival. Socio-economic status as a predictor of outcome was not assessed in this study. These are limitations of our study.

We assessed survival of our patients according to the Child-Pugh class. Assessing survival according to their MELD score would have been advantageous. The MELD score only came into wide use in 2002, when it was adopted as the criteria by which donor organs are distributed by UNOS (United Network for Organ Sharing) [6,30]. Our patients were followed up since 1995, and the MELD score at first presentation had not been calculated in a large proportion of our patients. However, Child Pugh class is still routinely used in clinical practice as a useful bedside assessment of patient prognosis and has been shown to be of similar prognostic value to MELD in some studies [31].

Ours was a retrospective study. A prospective study such as this would not have been possible in the current context because liver transplantation has commenced in our center in Sri Lanka.

We were able to contact 84.3% of the total registered population of cirrhotic patients. This represents the difficulties encountered during long term follow up of patients in a setting where hospitals and death registries are not linked electronically. In many cases, changes in addresses and phone numbers limited our ability to contact patients. Follow up of a large number of alcoholic cirrhotic patients whose compliance with clinic attendance is usually erratic is bound to be difficult in any setting.

## Conclusions

In a setting where liver transplantation is not readily available, we found that the overall survival of AC was similar to CC. Death in both groups were predominantly liver related, and was predicated by age at diagnosis and Child Pugh class, although survival was longer than that suggested in older studies. This may reflect better pharmacotherapy and endotherapy which is currently available altering the natural history of the disease. Among AC, those who continued to drink had the worst survival, and the presence of diabetes and non-abstinence from alcohol were independent predictors of mortality.

## Abbreviations

AC: Alcoholic cirrhosis; CC: Cryptogenic cirrhosis; MELD: Model For End-Stage Liver Disease.

## Competing interests

The authors declare that there are no competing interests.

## Authors’ contributions

HJdeS designed the research. SMS, MAN, SKW, JPdeA, APdeS, ASD, HJdeS carried out the research. AK annalized the data. SMS, MA and HJdeS wrote the final paper. All authors read and approved the final manuscript.

## Authors’ information

SMS (MBBS MD), Senior Registrar in Gastroenterology, University Medical Unit, Colombo North Teaching Hospital, Ragama

MAN (MBBS, MD, MRCP(UK), MRCP(Lond)), Consultant Gastroenterologist, University Medical Unit, Colombo North Teaching Hospital, Ragama

SKW(MBBS), Demonstrator, Department of Medicine, Faculty of Medicine, University of Kalaniya, Ragama,

AK (MBBS MD), Senior Lecturer, Department of Public Health, Faculty of Medicine, University of Kalaniya, Ragama,

JPdeA (MBBS), Demonstrator, Department of Medicine, Faculty of Medicine, University of Kalaniya, Ragama,

APdeS (MBBS, MD, MSc(Oxon), MRCP(UK), FRCP(Lond), FCCP), Professor in Medicine, Department of Medicine, Faculty of Medicine, University of Kalaniya, Ragama and Consultant Physician, University Medical Unit, Colombo North Teaching Hospital, Ragama

ASD (MBBS, MD, FCCP), Senior Lecturer, Department of Pharmacology, Faculty of Medicine, University of Kalaniya, Ragama,

HJdeS (MD, DPhil (Oxon), FRCP (Lond), FCCP, FNatAcadSci (SL), Hon. FRACP) Professor of Medicine, Department of Medicine, Faculty of Medicine, University of Kalaniya, Ragama and Consultant Physician, University Medical Unit, Colombo North Teaching Hospital, Ragama
